# Biomechanical and biochemical investigation of erythrocytes in late stage human leptospirosis

**DOI:** 10.1590/1414-431X20209268

**Published:** 2020-06-19

**Authors:** J.A.X. Silva, A.V.P. Albertini, C.S.M. Fonseca, D.C.N. Silva, V.C.O. Carvalho, V.L.M. Lima, A. Fontes, E.V.L. Costa, R.A. Nogueira

**Affiliations:** 1 Laboratório de Biofísica Teórico-Experimental e Computacional, Departamento de Morfologia e Fisiologia Animal, Universidade Federal Rural de Pernambuco, Dois Irmãos, Recife, PE, Brasil; 2 Centro de Apoio è Pesquisa, Universidade Federal Rural de Pernambuco, Dois Irmãos, Recife, PE, Brasil; 3 Laboratório de Química e Metabolismo de Lipídios e Lipoproteínas, Departamento de Bioquímica, Centro de Biociências, Universidade Federal de Pernambuco, Recife, PE, Brasil; 4 Colegiado de Ciências Biológicas, Universidade Federal do Vale do São Francisco, Petrolina, PE, Brasil; 5 Departamento de Biofísica e Radiobiologia, Universidade Federal de Pernambuco, Recife, PE, Brasil

**Keywords:** Leptospirosis, Lipid composition of membrane, Erythrocyte elasticity, Optical tweezers, Erythrocyte membrane

## Abstract

Leptospirosis is a zoonotic disease caused by bacteria of the genus *Leptospira*, which can cause lipid changes in the erythrocyte membrane. Optical tweezers were used to characterize rheological changes in erythrocytes from patients with leptospirosis in the late stage. Biochemical methods were also used for quantification of plasma lipid, erythrocyte membrane lipid, and evaluation of liver function. Our data showed that the mean elastic constant of erythrocytes from patients with leptospirosis was around 67% higher than the control (healthy individuals), indicating that patient’s erythrocytes were less elastic. In individuals with leptospirosis, several alterations in relation to control were observed in the plasma lipids, however, in the erythrocyte membrane, only phosphatidylcholine showed a significant difference compared to control, increasing around 41%. With respect to the evaluation of liver function of individuals with leptospirosis, there was a significant increase in levels of alanine transaminase (154%) and aspartate transaminase (150%), whereas albumin was 43.8% lower than control (P<0.01). The lecithin-cholesterol acyltransferase fractional activity was 3.6 times lower in individuals with leptospirosis than in the healthy individuals (P<0.01). The decrease of the erythrocyte elasticity may be related to the changes of erythrocyte membrane phospholipids composition caused by disturbances that occur during human leptospirosis, with phosphatidylcholine being a strong candidate in the erythrocyte rheological changes.

## Introduction

Leptospirosis is a bacterial disease caused by *Leptospira* species that are transmitted to human beings and animals through contact with soils and water contaminated with the urine of infected animals, predominantly rodents (1). This zoonosis occurs worldwide, but the incidence is highest in the tropical regions, being a disease with a great impact on the public health of the tropics (1–4). Leptospirosis is an infection with a broad geographical distribution due to the large spectrum of mammalian hosts that house and excrete the leptospires from their renal tubules (5,6). Outbreaks in tropical regions arise after heavy rainfall and flooding (1).

Leptospires are spirochetes that include saprophytic species (6 species with 60 serovars) and pathogenic species (13 species with more than 260 serovars) (7). Rodents are the main leptospirosis carriers, however, other wild or domestic animals can also be carriers, such as small marsupials, cattle, pigs, and dogs (3,7). The causative microorganism is capable of infecting the mammalian hosts through abraded skin and mucous membranes, and disseminating through the bloodstream to several organs (2).

Human leptospirosis presents clinical manifestations divided into two stages: the first stage or acute phase (septicemic phase) and the second stage or immune phase (4). The septicemic phase is characterized by fever, chills, headache, myalgia, skin rash, cough, anorexia, nausea, and vomiting (1,4,6). The immune or severe phase is known as Weil's disease and is characterized by jaundice, pulmonary hemorrhage, meningitis, liver, and acute kidney injury (1,3,6,7).

Deformability is defined as the matter capacity to change its shape in response to an applied force, which is a particularity of soft matter including red blood cells. Deformability can involve a change in cell curvature, a uniaxial deformation, or an area expansion (8). Thus, red blood cells are able to pass through capillaries with a smaller diameter than their size and to carry out their role as gas carriers between blood and tissues (8,9). Alterations in erythrocyte deformability have been associated with various diseases such as malaria, sickle cell anemia, diabetes, and hereditary disorders (9). Studies have shown that erythrocyte deformability is reduced in some diseases (10,11) and altered in patients with liver disease (12).

Cell deformability can be studied using optical tweezers (11,13,14). Optical tweezers are able to provide information on electrical and mechanical properties of red blood cells (13). Some studies have reported the use of optical tweezers to evaluate the erythrocyte elasticity as a way to investigate the deformability (11,13,14).

In this work, the optical tweezers were used to capture human erythrocytes and measure their elastic constant in patients in late stage leptospirosis. Biochemical techniques of identification and dosage (lipid composition of plasma and erythrocytes, some liver enzymes, and albumin) were used to establish possible relationships between lipid profiles of blood plasma and of the erythrocyte membrane with its elasticity.

## Material and Methods

### Collection and processing of blood samples

This study was approved by the Ethics Committee of the Hospital Universitário Oswaldo Cruz (HUOC), Recife, Brazil (CAAE-11524813.3.0000.5207). Blood samples were obtained from patients fasted for 12 h at HUOC. In a period of six months, only three patients presented the late stage of the disease (characterized by jaundice, respiratory failure, and kidney failure), for this reason just three blood samples were collected. Despite this, a great number of cells were analyzed. For control, 10 blood samples of healthy individuals were used. Peripheral blood was collected in vacuum tubes (Vacutainer, USA) containing anticoagulant EDTA-K^3+^ (1.8 mg/mL) to perform the biochemical assays. Additionally, blood was also collected in tubes without anticoagulant to obtain serum for the biomechanical experiment (assay with optical tweezers). The blood samples were processed over a period of up to 4 h and centrifuged at 2,500 *g* for 15 min at 4°C (Sorvall RC6, Thermo Fisher Scientific, USA). Then, a portion of blood was separated to evaluate erythrocyte elasticity. The remaining erythrocytes were washed 4 times with 4.5 mL of saline and again centrifuged at 2,500 *g* for 15 min at 4°C. The erythrocyte pellets were then used for lipid extraction.

### Measuring erythrocyte elasticity

The measurement of erythrocyte elasticity was carried out according to Moura et al. (13). The optical tweezers system consisted of a laser beam (λ=1064 nm, IPG Photonics, USA) focused on the microscope (Axio Lab, Carl Zeiss Microscopy GmbH, Germany) using a 100× objective lens, NA=1.25. The microscope was equipped with a motorized stage (Prior Scientific Instruments Ltd., UK) and an image capture system controlled by a computer. Cells from patients with leptospirosis (n=73 cells) and control individuals (n=222 cells) were captured by optical tweezers and dragged against blood serum at six different velocities (140 to 315 µm/s). The images of the erythrocytes deformation in the serum, according to the drag velocities, allowed us to measure their elastic constants. To determine the erythrocyte elasticity, the following equation was used (13):

$$∆L=\frac{ƞL_{0}^{2}}{µZ_{eq}}v$$

where ΔL = L *-* L_0_ is the cell length deformation (L_0_ is the length of the cell before dragging), η is serum viscosity that is measured by an Ostwald viscometer, μ is the elastic constant (also called apparent elasticity), and ν is the velocity. Z_eq_ is a parameter for the relative position of the erythrocyte to the Neubauer chamber, defined as follows: 1/Z_eq_ = 1/Z_1_ + 1/Z_2_. Z_1_ and Z_2_ are, respectively, the distance from the erythrocyte to the bottom and the top of a Neubauer chamber, in our case Z_eq_ = 25 μm. The value μ is calculated from a plot of cell length L *vs* the drag velocity ν, since the viscosity of the serum (η), the initial length (L_0_), and Z_eq_ are known. The cell length L was measured from the video images of the cells using Image-Pro Plus software (Media Cybernetics, USA).

### Lipid composition of plasma and erythrocyte membrane

Lipids were extracted from blood plasma and from erythrocyte membrane with chloroform:methanol (2:1, v/v), as described by Folch et al. (15) and Lima et al. (16). Aliquots of plasma and erythrocyte extracts were concentrated under a stream of N_2_. The isolation of the plasma phospholipids was performed by one-dimensional thin-layer chromatography with a solvent mixture containing chloroform:acetone:methanol:acetic acid:water (50:20:10:10:5, v/v). The isolation of the erythrocyte membrane phospholipids was performed by two-dimensional thin-layer chromatography on silica H containing 2.5% (w/w) of magnesium acetate. The mobile phase consisted, at the first dimension, of a solvent mixture containing chloroform:methanol:ammonia (65:35:5, v/v), and the second dimension was performed in a solvent system containing chloroform:acetone:methanol:acetic acid:water (50:20:10:10:5, v/v). Phosphatidylcholine (PC), sphingomyelin (SpM), phosphatidylethanolamine (PE), and lysophosphatidylcholine (LPC) were visualized after exposure to iodine vapor, labeled according to the relative mobility of the standards, and scraped from the silica tubes. These individual phospholipid samples were digested with 0.3 mL of 99.9% sulfuric acid by heating at 180°C for 2 h. The tubes were allowed to cool and after drops of 30% H_2_O_2_ were added, the tubes were again heated for 2 h (16,17). The inorganic phosphorus present in the phospholipids was quantified by the method of Bartlett (18). The total phospholipids were quantified from extracts of plasma and erythrocytes. Absorbance was measured spectrophotometrically at 735 nm.

The determination of triglyceride (TG), total cholesterol (TC), and high-density lipoprotein cholesterol (HDL-C) levels was performed by chemical and enzymatic methods using Labtest assay kit (Labtest Diagnostics Ltd., Brazil).

For the determination of triglycerides, Labtest kit used a lipoprotein lipase, glycerol kinase, glycerolphosphate oxidase, and peroxidase that act on the triglycerides of the sample, thus producing a quinoneimine (maximum absorbance at 505 nm). The concentration of triglycerides was determined from the absorbance ratio of the sample and standard solution.

Cholesterol was measured by the presence of antipyrilquinonimine. This product is obtained from the sample cholesterol by the action of cholesterol esterase, cholesterol oxidase, and peroxidase. After the formation of antipyrilquinonimine (maximum absorbance at 500 nm), it was quantified on the spectrophotometer.

HDL cholesterol was quantified in the supernatant after sample centrifugation. The colorimetric test, containing phosphotungstic acid and magnesium chloride, allowed the measurement of the endpoint reaction at 500 nm.

The levels of low-density lipoprotein cholesterol (LDL-C) and very-low-density lipoprotein cholesterol (VLDL-C) were determined by the Friedewald equation (19).

Liver function was evaluated measuring the alanine transaminase (ALT), aspartate transaminase (AST), albumin, and lecithin-cholesterol acyltransferase (LCAT) activity. AST, ALT, and albumin were measured with the Labtest assay kit (Labtest Diagnostics Ltd.).

The test for ALT is based on the catalytic action of ALT that transfers the alanine amino group to ketoglutarate, with the formation of glutamate and pyruvate. Pyruvate was reduced to lactate by lactate dehydrogenase, while coenzyme NADH was oxidized to NAD. As NADH coenzyme oxidation, monitored photometrically (reduction in absorbance at 340 nm), is directly proportional to ALT activity in the sample, it was possible to measure its concentration.

The test to measure AST is based on the catalytic action of AST, which transfers the amino group from aspartic acid to ketoglutarate, with the formation of glutamate and oxalacetate. Oxalacetate was reduced to malate by malate dehydrogenase, while coenzyme NADH was oxidized to NAD. The reduction in absorbance at 340 nm due to NADH coenzyme oxidation was monitored photometrically and is directly proportional to AST activity in the sample.

The measurement of albumin was done by binding albumin to bromocresol green dye. The color formed in the reaction was measured colorimetrically between 600 and 640 nm and it is proportional to the amount of albumin in the sample.

The LCAT activity was determined according to the method of Stokke and Norum (20), which uses a radioactive substrate. The substrate was prepared by adding 20 µL of (^14^C)-cholesterol (2 µCi) to a 5% (w/v) solution of human serum albumin in 1.0 mL of phosphate buffer saline (0.2 M), pH 7.4. The (^14^C)-free cholesterol and (^14^C)-esterified cholesterol were separated by thin-layer chromatography on silica G plates and removed from the chromatography plate to scintillation tubes. After the addition of scintillation fluid, the radioactivity of the samples was counted in the liquid scintillation counter Packard - Tri-Carb 2100TR (PerkinElmer Life Sciences, USA). The unit (U) of enzyme activity is reported as the percentage of free cholesterol (FC) converted to cholesterol ester (esterified cholesterol - EC) per hour.

### Statistical analysis

The biochemical data are reported as the mean±SE (standard error). The erythrocyte elasticity data are reported as the mean±SD (standard deviation). To analyze the biochemical data, Student's *t*-test was used and for erythrocyte elasticity data, a non-parametric Mann-Whitney test was used with a significance level of 5% (P=0.05).

## Results

### Erythrocyte elasticity

**Figure 1:**
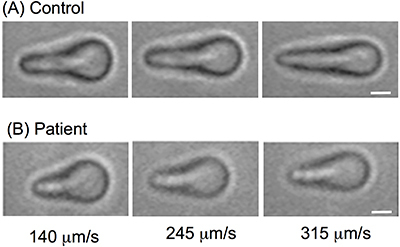
Representative erythrocyte elongations from controls (**A**) and patients with leptospirosis (**B**) obtained in experiments with optical tweezers. The dragging velocities used were 140, 245, and 315 µm/s. Scale bar: 2 µm.

The images were taken from a pool of cells from controls (n=222) and patients with leptospirosis (n=73). Figure 1A (erythrocytes from the control group individuals) and Figure 1B (erythrocytes from patients with leptospirosis) show typical erythrocyte elongations when dragged by the optical tweezer with velocities of 140, 245, and 315 µm/s. The different velocities are from the same sample. This figure shows that erythrocytes of the control individuals are more stretched out than the patients.

**Figure 2:**
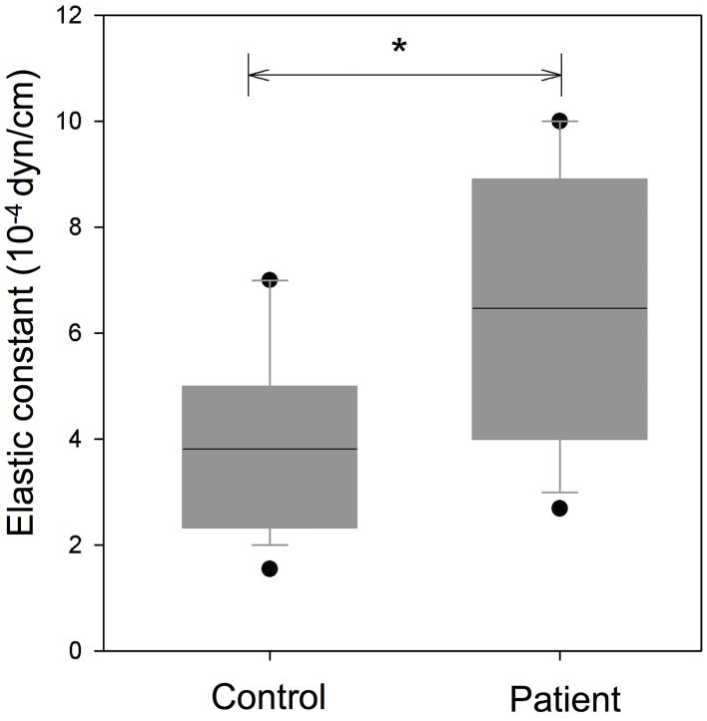
Box plot of erythrocyte elasticity for controls and patients with leptospirosis. The black lines represent the median of the elastic constant of erythrocyte for control and patients with leptospirosis. *P<0.05 (Mann-Whitney test).

Figure 2 shows the boxplot of elasticities for patient and control erythrocytes. The optical tweezers experiments showed that the average values of erythrocyte elastic constant obtained from patients and healthy individuals were (6.5±2.5)×10^-4^ dyn/cm and (3.9±1.9)×10^-4^ dyn/cm, respectively. Erythrocyte elastic constants were statistically different (P<0.05) between patients with leptospirosis and healthy individuals. The value of the erythrocyte elastic constant is inversely related to its stretching length. Under the analysis of optical tweezers, when the cell stretching length is longer, the value of the elastic constant is smaller.

The results showed that the red blood cells of leptospirosis patients had higher elastic constant values and, thus, a shorter stretch length. In other words, elasticity was reduced in diseased red blood cells.

### Lipid content of plasma and erythrocyte membrane

Table 1 shows the lipid composition of blood plasma and erythrocyte membrane of healthy individuals and patients with leptospirosis. Patients with leptospirosis exhibited a reduction of 40.1% for TC, 58.0% for HDL-C, and 69.4% for LDL-C compared to the control group. TG and VLDL-C were higher in patients with leptospirosis, with an increase of 88.4 and 130%, respectively, in relation to the control. Moreover, the ratio of TC/HDL-C showed an increase of 42.8% in patients with leptospirosis.

**Table 1:** Lipid composition of plasma and erythrocyte membrane and biochemical analysis of liver function of control subjects and patients with leptospirosis.

	Controls (n=10)	Patients (n=3)	P
Plasma			
TC (mg/dL)	172.40±27.96	103.33±9.26	0.0018
HDL-C (mg/dL)	41.63±7.83	17.47±3.89	0.0004
LDL-C (mg/dL)	101.69±19.90	31.13±14.35	0.0002
VLDL-C (mg/dL)	23.77±10.15	54.80±7.03	0.0009
TG (mg/dL)	145.40±40.94	274.00±35.17	0.0002
PC (%)	64.62±2.05	70.55±0.67	0.0001
PE (%)	7.60±0.58	15.46±0.39	0.0001
SpM (%)	19.34±1.05	9.37±0.09	0.0001
LPC (%)	8.45±0.01	6.63±0.05	NS
FC (%)#	20.90±2.93	38.03±4.40	0.005
EC (%)#	79.10±2.95	61.97±4.45	0.0051
Erythrocyte membranes			
PC (%)	33.43±7.25	47.06±4.74	0.0117
PE (%)	31.31±11.02	27.40±1.75	NS
SpM (%)	28.48±6.21	20.66±2.86	NS
LPC (%)	6.77±2.36	4.88±1.25	NS
Liver-specific analysis			
Albumin (g/dL)	4.50±0.29	2.53±0.30	0.0001
ALT (U/L)	25.20±10.28	64.00±17.05	0.0004
AST (U/L)	23.90±4.07	59.67±15.53	0.0001

Data are reported as mean±SD. Student's *t*-test was used. TC: total cholesterol; HDL-C: high density lipoprotein cholesterol; LDL-C: low density lipoprotein cholesterol; VLDL-C: very low density lipoprotein cholesterol, TG: triglycerides: PC: phosphatidylcholine; PE: phosphatidylethanolamine, SpM: sphingomyelin; LPC: lysophosphatidylcholine; FC: free cholesterol; EC: esterified cholesterol; ALT: alanine transaminase; AST: aspartate transaminase; NS: not significant. #n=3 for controls and patients for FC and EC.

Patients with leptospirosis presented a rise compared to the control group of 9.2% for plasma PC, 40.8% for erythrocyte membrane PC, and 103.4% for plasma PE. On the other hand, the plasma SpM of patients was 51.6% lower than the control. The plasma and erythrocyte membrane LPC values did not show a significant difference between groups, as well as the PE and SpM of the erythrocyte membrane.

### Biochemical data of liver function

In individuals with leptospirosis, a significant increase in levels of ALT (154%) and AST (150%) were found compared to the control group. The increased level of these two liver enzymes is related to liver dysfunction.

The albumin level of patients was 43.8% lower compared to the control group (P=0.0001), which can indicate an abnormality in liver function.

The fractional LCAT activity was 3.6 times lower in individuals with leptospirosis than in healthy individuals (control) (P<0.0001). In individuals with leptospirosis, an activity of 0.11±0.07 U was obtained, compared to 0.40±0.03 U in the control group. FC was 20.9% in the control group and 38.03% in the patients, whereas EC was 79.1% and 61.97%, respectively. The values mentioned above indicated that a decrease of the LCAT activity promoted an increase in free cholesterol in patients with leptospirosis.

## Discussion

The deformability of erythrocytes plays an essential role in the transport of gases via blood vessels (8,9,11,13,14). It is a rheological property related to viscoelasticity of both the cell membrane and cytosol that can be altered by certain diseases (10). Garnier et al. (10) used the Hanss hemorheometer to measure erythrocyte deformability and observed a higher index of rigidity in diabetic patient’s samples, whilst Agrawal et al. (11) observed a reduction of deformability in diabetic patient’s erythrocytes using optical tweezers.

Optical tweezers can be used for investigating red blood cell rheology. Our study showed that the erythrocyte elastic constant of patients with leptospirosis was higher compared to the control indicating that their erythrocytes were more rigid than the cells of healthy individuals. Santoro et al. (21) observed a lower erythrocyte osmotic fragility in dogs with *Leptospira interrogans*, which suggests higher erythrocyte rigidity, or lesser deformability, than the control (21). A decrease in the erythrocyte deformability causes a significant increase in microvascular flow resistance and blood viscosity (8).

The TG level was increased for patients with leptospirosis, and this result was compatible with data reported by Peces (22), Liberopoulos et al. (23), and Gazi et al. (24). The levels of LDL-C and TC were decreased, confirming the results described by Liberopoulos et al. (23) and Gazi et al. (24). These authors have shown a correlation, direct or inverse, between lipids and lipoproteins and cytokine concentrations. Liberopoulos et al. (23) described an increase of interleukin-6 (IL-6) and tumor necrosis factor-α (TNF-α) in severe leptospirosis. IL-6 is capable of stimulating the expression of genes responsible for LDL receptors, decreasing LDL-C, while TNF-α stimulates hepatic TG synthesis. According to Liberopoulos et al. (23), IL-6 is responsible for the reduction of TC and this reduction was also observed in our study. Various inflammatory cytokines (IL-1β, IL-6, IL-12, TNF-α) are increased in leptospirosis as an immune response to the infection, however, this increase may be higher in severe stages of the disease (25).

Vanaja et al. (26) observed an elevation of TG, VLDL, and phospholipids, and a reduction of HDL in young guinea pigs infected by *Leptospira interrogans* serovars *australis, canicola*, and *icterohaemorrhagiae*. These authors also noted an increasing trend of cholesterol and LDL for pigs with serovar *icterohaemorrhagiae*. Gazi et al. (24) and Cisternas and Milstein-Kuschnaroff (27 [Bibr B28]) have also reported that the HDL-C level is reduced, whereas the VLDL-C level is increased in patients with leptospirosis, as we have observed. Cisternas and Milstein-Kuschnaroff (27) have reported the LDL-C level to be decreased.

Changes in liver function promoted by leptospirosis are normally found in the severe phase (1,3,6,7). Liver injury caused by leptospirosis implicates in modifications of biochemistry parameters as ALT and AST; normally both enzymes are increased in the course of the disease (29) and our results were in agreement.

Albumin is the most abundant protein in plasma and predominantly synthesized in the liver (29,30). Its reduced values, as observed in this study, mean alterations in liver function (29). In the advanced stage of leptospirosis, as the liver becomes the target of this disease, changes in the synthesis of liver enzymes and proteins are expected. Gancheva (30) reported hypoalbuminemia in leptospirosis, in addition to hypoproteinemia, and elevated ALT and AST values.

In liver disease, there is an alteration in the erythrocyte membrane lipid composition that is associated with abnormalities in the composition of plasma lipoproteins. Such changes can lead to modifications in the erythrocyte shape and deformability (12). We observed that plasma levels of PC and PE were increased whilst SpM was decreased in patients with leptospirosis. Meanwhile, we did not observe a significant difference in the values of PE and SpM in erythrocyte membrane. Owen et al. (12) reported an increase in the PC fraction in erythrocyte membrane of patients with liver disease, while the proportion of PE and SpM were reduced in these patients compared to normal subjects. Furthermore, we did not identify differences in the LPC levels in plasma and erythrocyte membrane of patients with leptospirosis.

Shiraishi et al. (31) reported a decrease in erythrocyte deformability and erythrocyte membrane fluidity, abnormal lipid compositions, and increased PC:SpM ratio in patients with alcoholic liver disease. The lipid domain fluidity of the erythrocyte membrane is determined by the molar ratio of cholesterol to phospholipid, degree of unsaturation of phospholipid acyl chains, and the PC/SpM ratio (32). Our results showed that the phospholipid constitution of the erythrocyte membrane of patients with leptospirosis was not significantly altered, except for PC, whose mean value was 40.8% higher in patients than controls. Thus, the increase of PC seemed to contribute to the decrease of erythrocyte deformability, while there was no significant alteration for other phospholipids. However, according to Borochov et al. (33), PC forms highly fluid lipid regions, while sphingomyelin induces rigidity. Kuypers et al. (32) showed that changes in the molecular species composition of PC alter the morphology and erythrocyte deformability. The replacement of native PC with 1-palmitoyl, 2-arachidonoyl PC resulted in lower osmotic fragility, whereas replacement with 1,2-dipalmitoyl PC led to higher osmotic fragility.

LCAT is an enzyme synthesized by the liver that plays a part in the cholesterol transport process by converting FC to EC to form mature HDL (34). EC generated by LCAT is more hydrophobic than FC and, thus, migrates into the hydrophobic core of the lipoprotein, resulting in the conversion of pre-β-HDL into α-HDL (35). We observed a fractional LCAT activity reduction in patients with leptospirosis. Cisternas and Milstein-Kuschnaroff (27) also reported a reduction in fractional LCAT rate in patients with leptospirosis in relation to the control. LCAT activity, as well as the concentration of this molecule, is directly proportional to the HDL-C levels (35). Therefore, the decrease of enzyme activity is associated with the HDL-C reduction, since LCAT is present in the HDL (34,35). This process of cholesterol esterification in plasma by LCAT is essential for cholesterol uptake from the liver. A large amount of EC formed by LCAT is exchanged with triglycerides through the mediated process by cholesteryl ester transfer protein into apolipoprotein B containing lipoproteins that are finally catabolized by the liver. Alternatively, HDL-cholesteryl esters are taken-up by the liver through scavenger receptor class B member 1 (35,36). This is the clearance process of FC from plasma lipoproteins. Our study showed a reduction of LCAT activity that reflected in a decrease of EC levels in patients with leptospirosis, resulting in an increase in FC.

Summarizing our results, the optical tweezers showed that erythrocyte elasticity was modified by leptospirosis. Also, leptospirosis promoted lipid concentration changes in plasma. However, in the erythrocyte membrane, only PC was increased, which probably had a contribution in the decrease of erythrocyte elasticity.
